# Grit and Second Language Learning Engagement: The Mediating Role of Affect Balance

**DOI:** 10.3390/bs14030184

**Published:** 2024-02-26

**Authors:** Chenggang Wu, Xiaoyong Tian, Hui Jin

**Affiliations:** 1 Key Laboratory of Multilingual Education with AI, School of Education, Shanghai International Studies University, Shanghai 201620, China; chenggangwu@outlook.com (C.W.); jinhui@shisu.edu.cn (H.J.); 2 Institute of Linguistics, Shanghai International Studies University, Shanghai 201620, China

**Keywords:** grit, positive emotions, negative emotions, affect balance, learning engagement

## Abstract

The study of the relationship between key psychological attributes of learners and their engagement in second language (L2) learning helps to understand the critical personality mechanisms influencing language learning. The present study examined the L2 learning engagement from the perspectives of grit (i.e., consistent efforts and interests devoted to a long-term goal) and affect balance (a notion that takes into account both positive and negative emotions concurrently, assessing and evaluating which side holds more significance or influence). A cohort of English L2 learners (*N* = 394) participated in an online survey aimed at gauging their levels of grit, affect balance, and engagement in L2 learning. The results indicated that grit and affect balance were significantly correlated with behavioral engagement and affective engagement in L2 learning. However, among the two components of grit, namely consistency of interest, showed no significant relationship with L2 learning engagement, while perseverance of effort was significantly positively correlated with L2 learning engagement. Affect balance played a partially mediating and full mediating role between perseverance of effort and behavioral engagement as well as affective engagement respectively. These findings confirm the crucial role of perseverance of effort in second language learning and reveal the unique role of affect balance in their relationship.

## 1. Introduction

In recent years, research in the field of second language acquisition has continuously intersected with psychological studies. New perspectives have been drawn from personality psychology [1] and positive psychology [2]. Various aspects of the mechanisms influencing language learning are explored by examining internal and external influencing factors on learners. The research related to positive psychology has become a hot topic in foreign language learning [2,3,4,5]. Positive psychology, breaking free from the previous emphasis on how negative psychology influences language learning, focuses on the positive aspects of human nature, such as grit [6], enthusiasm [4], hope [7], and more. Grit, as a concept at the intersection of personality psychology and positive psychology, has received sustained attention in recent second language (L2) studies [8,9].

### 1.1. Grit and L2 Learning

Grit refers to an individual’s “consistent perseverance and interest towards long-term goals” [10,11]. Therefore, Duckworth et al. [10] divided grit into two components: perseverance of effort (PoE) and consistency of interest (CoI). PoE refers to an individual’s ability to persist in doing something uninterruptedly, even in the face of setbacks. CoI is the unwavering passion for something over time, rather than a fleeting interest followed by a change in focus. Research has validated that grit consists of both PoE and CoI [11], but subsequent studies found that it is PoE, not CoI, that significantly predicts academic achievement [12]. This suggests that, in the academic context, PoE in grit may play a more important role than CoI [13].

Recent studies have pointed out the need to design a specific grit scale for second language (L2) learning due to its uniqueness [14]. Teimouri et al. [14] designed and validated an L2 grit scale based on Duckworth et al. [11]. The primary distinction between general grit and L2 grit lies in the focus of measurement: while general grit assesses perseverance and passion for long-term goals in various domains, L2 grit specifically evaluates these qualities within the context of second language (L2) learning. For instance, a typical item in general grit, such as “I am diligent”, gauges perseverance of effort (PoE) across different areas. In contrast, an item in L2 grit would be modified to reflect the specific context, such as “I am a diligent English language learner”, emphasizing perseverance and passion specifically within the realm of L2 acquisition. Overall, the results show that L2 grit is positively correlated with learners’ language learning motivation and academic achievement, and this correlation is stronger than the results obtained using a general grit scale. Subsequent research using the L2 grit scale found positive correlations between L2 grit and L2 enjoyment and proficiency [15], positive correlations between L2 grit and L2 listening and reading self-efficacy, and negative correlations with L2 reading and listening anxiety [16]. Khajavy et al. [17] found that general grit could not significantly predict L2 academic achievement. These results indicate that the level of grit measured by a general grit scale does not have a significant relationship with L2 learning, unlike L2-specific grit [17]. However, some studies have found a negative correlation between general grit and L2 learning anxiety [6], suggesting that general grit can predict L2 academic performance by influencing L2 enjoyment [15]. Recent findings have further substantiated the strong link between enjoyment in L2 learning and general grit. For instance, in a study by Lee [18], it was discovered that perseverance of effort (PoE) exhibited a positive correlation with enjoyment in L2 learning across middle school, high school, and university students. However, while there was a positive correlation between classroom enjoyment and CoI for middle and high school students, this association was not observed among university students. Lee [18] also found that PoE in general grit could significantly predict L2 communication willingness, while CoI could not predict L2 communication willingness, and this result was also consistent across middle school, high school, and college students [18].

Although some studies use the L2 grit scale and others use a general grit scale, consistently, research results show a significant correlation between grit and L2 learning. It is worth noting that most existing studies demonstrating the effective prediction of L2 learning-related variables by general grit are based on L2 learner groups in China. Therefore, this study continues to use a general grit scale to measure the level of PoE and CoI in L2 learners. In addition, despite abundant evidence indicating a significant relationship between grit and L2 academic emotions [15,18], these studies lack a systematic understanding of emotions, especially a balanced understanding of positive and negative emotions. To delve deeper into this matter, we would introduce L2 emotion research, highlighting its evolution from a negative focus to a positive focus in the subsequent section. Additionally, we will explore the potential for jointly considering both positive and negative affects in this context.

### 1.2. L2 Academic Emotions: From Negative to Positive and Then to Balance

In the field of applied linguistics, there has been a noticeable shift towards emotions in the last 20 years, recognizing the limitations of previous research that focused too much on cognitive processes [19]. Early research on language learning emotions mostly concentrated on negative emotions, particularly language learning anxiety [20]. MacIntyre [20] summarized that language anxiety could lead to a decrease in academic performance, a decrease in perceived L2 abilities, a decrease in learners’ willingness to communicate, and interference in cognitive processes such as language input, processing, and production. A recent meta-analysis found a significant negative correlation (*r* = −0.36) between L2 anxiety and L2 academic achievement. Moreover, L2 anxiety can explain 13% of the variance in L2 academic achievement [21], indicating that L2 emotions may affect L2 academic achievement.

Recent research has shifted from the perspective of negative emotions to positive emotions. For instance, Dewaele and MacIntyre [22] first introduced the variable of foreign language enjoyment (FLE) and found a significant negative correlation between FLE and language learning anxiety. Recent research further found that positive emotions play a mediating role in the relationship between teacher support and students’ engagement in foreign language learning, and this mediating role is equally significant for both males and females [23]. In addition to enjoyment, other positive emotions such as passion and hope have also gained attention in L2 learning. For example, Chen et al. [24] found that harmonious passion is more predictive of positive performance in L2 learning activities, compared to obsessive passion. Furthermore, enthusiasm for L2 learning can transfer to other domains and promote personal growth. These results indicate that positive emotions from the perspective of positive psychology play a positive role in L2 learning, supporting a more complete depiction of the panorama of L2 learning emotions.

The application of positive psychology in L2 learning is not a disregard for defects and negative emotions but a search for balance between positive and negative emotions in humans. This perspective provides a new angle for the study of L2 learning emotions, namely affect balance. Affect balance is a concept that simultaneously considers positive and negative emotions, comparing and weighing which side has greater weight. It is often calculated by subtracting the score of negative emotions from the score of positive emotions [25]. Individuals with higher affect balance scores generally have more positive emotions and fewer negative emotions. Some studies also indicate that higher affect balance is associated with less emotional variability, while lower affect balance is associated with higher nightly stress [26]. This suggests that affect balance, as a component of subjective well-being, is closely related to an individual’s life satisfaction [26]. Emerging from the positive psychology movement in L2 learning, there has been notable value in exploring how emotions influence L2 learning from an affect balance perspective. This approach enhances previous perspectives, which tended to be either positive dominant or negative dominant, by embracing a balanced understanding that incorporates both negative and positive affects.

### 1.3. L2 Learning Engagement: The Relationship between Grit and Academic Emotions

Research on learning engagement has accumulated significantly in the field of learning science, but there has been relatively less focus on it in the context of L2 learning [27]. Generally, in L2 learning, learning engagement is considered to be the effort exerted by L2 learners in the learning process, including various elements such as behavior and emotions [27]. Some studies have found that L2 learning anxiety has a negative impact on learning engagement [28]. Teacher support can positively predict L2 learning engagement [29] and positive academic emotions play a mediating role in this positive predictive effect [23]. It can be seen that recent research in L2 learning has begun to explore the correlation between L2 learning emotions and engagement, obtaining reliable relationship validations. However, the relationship between L2 grit and L2 learning engagement has not been verified. Liu [8] briefly reviewed the relevant studies on L2 learning engagement and grit, suggesting a possible positive relationship between the two, but did not provide supporting data [8]. As language learning often involves a challenging and prolonged journey marked by numerous obstacles and setbacks, learners must cultivate a positive mindset to navigate these challenges effectively and maintain their engagement with the learning process. Grit, characterized by steadfast commitment and perseverance toward long-term goals, emerges as a crucial trait in empowering language learners to overcome obstacles and sustain their engagement over time [8]. Therefore, this study attempts to simultaneously measure grit and L2 learning engagement and explore the relationship between the two. Learning engagement is a layered construct, including behavioral, affective, cognitive, and agentic engagement [27], and the former two were widely explored in the literature [30]. Since the present study was an initial attempt to investigate the relationship between engagement and affect balance in the L2 learning context, behavioral and affective engagements were its focus.

### 1.4. The Present Study

Despite the growing attention to L2 grit, current research results are controversial, especially regarding the unclear relationship between CoI and L2 learning. Most results suggest that the consistency of interest (CoI) within grit cannot positively predict L2 academic achievement [31]. However, there is also evidence indicating that CoI can predict L2 learning emotions. Some studies have not distinguished between the two dimensions of grit but instead treated them as a whole when studying their relationship with L2 learning [15]. Given the inconsistency in existing research and different measurement approaches, this study, assuming grit as a whole, further differentiates CoI and perseverance of effort (PoE) to investigate the relationship between grit and L2 learning engagement. Additionally, current L2 emotion research lacks the perspective of affect balance, usually distinguishing positive and negative emotions, which is not conducive to exploring the comprehensive characteristics of individual learning emotions. This study measures both negative and positive emotions in L2 learning, calculates affect balance, and explores the relationship between L2 affect balance and L2 learning engagement.

Recently, Liu and Wang [9] found that in the relationship between grit and foreign language performance, foreign language enjoyment, and anxiety act as mediating variables influencing the relationship between grit and language performance, with a stronger mediating effect of foreign language anxiety. However, Liu and Wang [9] did not investigate the impact of affect balance on the relationship between grit and foreign language learning engagement. When considering affect balance, which entails integrating positive emotions alongside negative ones, a more holistic comprehension of emotional experiences emerges. Based on the above analysis, the research questions for this study are as follows:

RQ1: How are grit (PoE and CoI), L2 affect balance, and L2 learning engagement related?
RQ2: How do grit (PoE and CoI) and L2 affect balance predict L2 learning engagement?
RQ3: Does L2 affect balance have a mediating effect in the relationship between grit and L2 learning engagement?

## 2. Method

### 2.1. Participant

Convenience sampling was adopted. A total of 394 (367 freshman and 27 sophomores) participated in the study, including 100 males, with an overall average age of 20.19 years. The students were learning English as their second language and had been learning English since primary education. The participants spend 3–4 h per week on English language learning.

### 2.2. Measurement

#### 2.2.1. Grit

The grit scale was initially proposed by Duckworth et al. [11], and later Duckworth and Quinn [10] introduced a shortened version of the grit scale, consisting of eight items that measure perseverance of effort (PoE) with four questions and consistency of interest (CoI) with four questions. The scale uses a 5-point rating, ranging from 1 (“Not at all like me”) to 5 (“Very much like me”). This scale has been used in previous studies [6,15]. In this study, the internal consistency reliability (Cronbach’s alpha) for PoE and CoI on this scale were 0.76 and 0.75, respectively. The confirmatory factor analysis provided construct validity for grit (CFI: 0.98, NFI: 0.98, IFI: 0.98, SRMR: 0.08)

#### 2.2.2. Affect Balance

The affect balance questionnaire was adapted from Diener et al.’s [25] research, measuring individuals’ emotional experiences in engaging in certain activities over a recent period. In this study, it inquired about students’ emotional experiences in learning English over the past three months, using a 5-point scale ranging from “Very rarely or never” to “Very often or always”. The chosen emotional descriptors included “Positive”, “Satisfied”, “Good”, “Happy”, “Enjoyable”, “Joyful”, “Sad”, “Unpleasant”, “Worried”, “Bad”, “Negative”, and “Angry”. Positive emotions were calculated by summing the scores of the first six emotions, while negative emotions were calculated by summing the scores of the latter six emotions. Affect balance was derived by subtracting the negative emotion score from the positive emotion score. The internal consistency reliability (Cronbach’s alpha) for the positive emotion questionnaire and negative emotion questionnaire in this study were 0.92 and 0.89, respectively. The construct validity was also confirmed (CFI: 0.99, NFI: 0.99, IFI: 0.99, SRMR: 0.05).

#### 2.2.3. Language Engagement

The learning engagement questionnaire was adapted from Skinner et al.’s [30] wording has been altered to cater to English learning context, covering both behavioral (e.g., “I strive to achieve good grades in my English class”) and affective aspects (e.g., “I feel very good during English class”) in English learning context of students’ learning engagement with five items each. Behavioral engagement assesses actions dedicated to English learning, while affective engagement evaluates positive sentiments and attitudes toward English learning. A 7-point scale was used, with response options ranging from 1 (“Extremely unlike me”) to 7 (“Extremely like me”). In this study, the internal consistency reliability (Cronbach’s alpha) for behavioral engagement and affective engagement were 0.90 and 0.94, respectively. The confirmatory factor analysis showed learning engagement had a good model fit (CFI: 0.99, NFI: 0.99, IFI: 0.99, SRMR: 0.07).

## 3. Results

### 3.1. Descriptive Statistics and Correlation Analysis

Table 1 presents the descriptive statistics of the variables. It is apparent that students exhibit a relatively high level of L2 learning engagement, as evidenced by a mean score of 5 out of 7, and their grit is also at a moderately medium level (2.59/5). Regarding learning emotions, both positive and negative emotions are present. Therefore, the majority of students are in a relatively balanced affective state.

From the results of the correlation analysis (Table 2), it is evident that there was no significant relationship between consistency of interest (CoI) and L2 learning engagement, including both behavioral and affective engagement. Additionally, positive L2 learning emotions did not show a significant correlation with CoI. However, the remaining variables exhibited significant correlations. Specifically, negative academic emotions showed a negative correlation with learning engagement, while positive learning emotions and grit were significantly positively correlated with learning engagement.

### 3.2. Predictive Effects of Grit and Affect Balance on Learning Engagement

Grit and affect balance were entered into a regression model as predictor variables for learning engagement. There was no apparent linear covariation between the two predictor variables, as indicated by VIF values less than 1.12. The model showed good explanatory power with an *R*^2^ of 0.46, and *p* < 0.001. However, grit did not significantly predict L2 behavioral engagement (*β* = 0.08, *t* = 1.64, *p* > 0.1), while affect balance (*β* = 0.43, *t* = 9.22, *p* < 0.001) was a significant predictor for L2 behavioral engagement. For affective engagement, affect balance as a predictor variable also had a significant predictive effect, with an *R*^2^ of 0.63, *p* < 0.001, and *β* = 0.63, *t* = 15.35, *p* < 0.001. Grit, on the other hand, could not significantly predict affective engagement (*β* = −0.02, *t* = −0.45, *p* > 0.1).

Since consistency of interest (CoI) did not show a significant relationship with learning engagement, further analysis was conducted to explore whether the weaker predictive effect of grit on behavioral and affective engagement was due to simultaneously considering CoI and PoE. PoE and affect balance were included in the regression model as predictors for learning engagement. For behavioral engagement, both PoE (*β* = 0.22, *t* = 4.74, *p* < 0.001) and affect balance (*β* = 0.38, *t* = 8.28, *p* < 0.001) independently and significantly predicted behavioral engagement, with an *R*^2^ of 0.50, *p* < 0.001. However, PoE could not significantly predict affective engagement in L2 learning (*β* = 0.08, *t* = 1.93, *p* > 0.05), while affect balance could significantly predict affective engagement (*β* = 0.60, *t* = 14.48, *p* < 0.001).

### 3.3. The Mediating Role of L2 Affect Balance in the Relationship between Grit and L2 Learning Engagement

**Figure 1:**
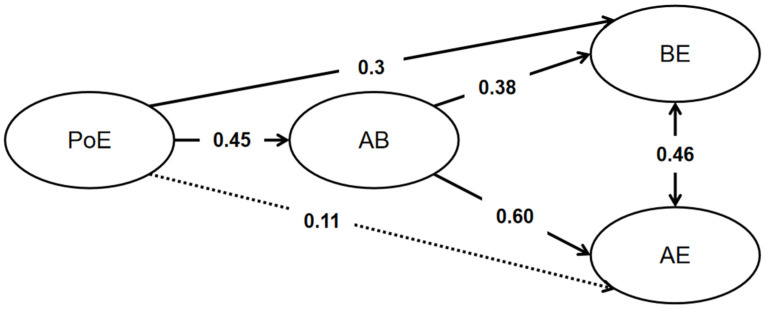
The mediating role of affect balance (AB) in the relationship between PoE (perseverance of effort) and L2 behavioral (BE) and affective engagement (AE). The dotted lines indicate the non-significance (*p* > 0.05).

Due to the relatively weak correlation between consistency of interest (CoI) and L2 learning engagement, the focus here was primarily on the relationship between perseverance of effort (PoE) and L2 learning engagement. The specific results of the mediation model are illustrated in Figure 1. PoE could directly predict behavioral engagement (*β* = 0.30, *z* = 4.76, *p* < 0.001) but only marginally predicted affective engagement (*β* = 0.11, *z* = 1.94, *p* = 0.053). However, PoE could predict both behavioral engagement (*β* = 0.17, *z* = 5.38, *p* < 0.001) and affective engagement (*β* = 0.27, *z* = 6.35, *p* < 0.001) through the mediating effect of affect balance. These results indicated that the relationship between grit and both behavioral and affective engagement in L2 learning was influenced by affect balance. The relationship between grit and behavioral engagement was partially mediated by affect balance, while the relationship between grit and affective engagement was fully mediated by affect balance.

## 4. Discussion

The present study, for the first time, investigated the predictive roles of grit and affect balance on L2 learning engagement. The results indicated that, overall, both the PoE and affect balance were correlated with and significantly predicted L2 behavioral engagement rather than affective engagement. Additionally, in the relationship between PoE and L2 learning engagement, affect balance played a significant mediating role.

### 4.1. The Correlation between Grit and L2 Affect Balance

The correlational analysis in this study revealed a significant association between grit and L2 positive emotions, negative emotions, and affect balance. These findings align with previous research; for instance, Teimouri et al. [14] found significant correlations between general grit and language enjoyment (*r* = 0.21, *p* < 0.001) and anxiety (*r* = −0.26, *p* < 0.001). Additionally, Teimouri et al. [14] used an L2 grit scale and found a significant correlation between L2 grit and language enjoyment (*r* = 0.45, *p* < 0.001) and a negative correlation with language anxiety (*r* = −0.27, *p* < 0.001). Li and Dewaele [6] found that general grit could significantly predict foreign language anxiety. Similar to Li and Dewaele [6], this study also found that general grit could significantly predict affect balance (*β* = 0.31, *t* = 6.40, *p* < 0.001). These consistent findings affirm the crucial role of general grit in L2 learning, especially in influencing emotional reactions during the language learning process. High levels of grit may aid individuals in coping with negative emotions, such as anxiety. However, further research is needed to explore the relationship between grit and other negative emotions, such as shame and confusion.

### 4.2. Grit and L2 Learning Engagement: PoE Rather Than CoI Plays a Role

The results of this study indicate that general grit cannot significantly predict individuals’ L2 learning engagement, including behavioral and affective engagement. These results suggest that the impact of general grit on L2 learning engagement is limited, and a crucial reason for this limitation is that general grit encompasses both PoE and CoI. These two components show differential relationships with learning engagement. Correlational results indicate a significant positive correlation between PoE and L2 learning engagement, including both behavioral and affective engagement. However, CoI shows no correlation with either behavioral or affective engagement. These findings are similar to those of Teimouri et al. [14], who found that PoE had a significant correlation with grammar scores, speaking scores, GPA, and self-reported language proficiency, while CoI did not show significant correlations. These results imply that, compared to CoI, PoE has a closer relationship with language learning. As the difficulty of language learning increases and learning time extends, the motivation to maintain higher levels of learning engagement may be driven more by the inertia of PoE than by CoI. Over time, many language learners may lose their initial interest and passion but still require sustained effort to achieve a certain language proficiency level. Therefore, individuals with high levels of grit are more likely to adopt persistent effort to achieve their goals.

These findings also highlight the criticism of measuring general grit, including both PoE and CoI [32]. PoE, compared to general grit, shows a stronger correlation with L2 learning. This indicates that, when analyzing grit as a personality trait, it is essential to consider the differential contributions of its various aspects. Therefore, in future research, when analyzing grit as a personality trait, more attention should be paid to PoE rather than CoI. However, PoE cannot significantly predict L2 affective engagement, indicating that the personality trait of grit may have a more substantial impact on language learning behavior than on affective engagement. One possible explanation for the finding that general grit cannot predict L2 affective engagement may be that general grit may not be as evident in L2 learning as L2 grit. There is evidence suggesting that L2 grit and general grit produce different results concerning their correlation with language learning achievement. For example, L2 grit is significantly correlated with grammar scores (*r* = 0.26, *p* < 0.001), speaking scores (*r* = 0.30, *p* < 0.001), GPA (*r* = 0.45, *p* < 0.001), and self-evaluated language proficiency (*r* = 0.31, *p* < 0.001). In contrast, general grit shows no significant correlation with grammar scores (*r* = 0.05, *p* > 0.05) and self-evaluated proficiency (*r* = 0.11, *p* > 0.05), and weaker correlations with speaking scores (*r* = 0.18, *p* < 0.05) and GPA (*r* = 0.14, *p* < 0.05) [14]. These results suggest that L2 grit may be more relevant to L2 academic performance. However, general grit is still correlated with various L2 academic achievements, albeit to a lesser extent [14]. Additionally, the reliability coefficient of CoI in the L2 grit scale designed by Teimouri et al. [14] is only 0.66, slightly lower than the desirable reliability level of 0.7. Therefore, the study provides corrected correlation coefficients when conducting correlational analysis [14]. In contrast, the general grit scale shows high reliability coefficients, all exceeding 0.75. Therefore, there is room for improvement in the reliability of L2 grit in that study, which may affect the stability of the correlation results. Nevertheless, the general grit scale’s high reliability coefficients and its correlations with relevant language learning emotions and academic achievements confirm that general grit can be still used in L2 learning research.

### 4.3. Affect Balance as a Mediator in the Relationship between Grit and L2 Learning Engagement

Another contribution of this study is the examination of L2 learning emotions’ predictive role and their mediating effect on the relationship between grit and L2 learning engagement from the perspective of affect balance. Affect balance, considering both positive and negative emotions, has been shown to be closely related to life satisfaction [33]. This study also found a significant correlation between L2 affect balance and L2 learning engagement, including both behavioral and affective aspects. Compared to positive emotions, the correlation strength between L2 affect balance and engagement is weaker, indicating that pure positive emotions play a stronger role in L2 learning engagement. However, L2 affect balance, by incorporating positive emotions after accounting for negative emotions, provides a more comprehensive understanding of emotional experiences. As individuals experience various emotions, including both positive and negative, examining only positive or negative emotions cannot capture a balanced understanding of emotions. Some perspectives suggest that excessive positive emotions can also bring negative impacts, and negative emotions have adaptive functions [34]. For example, if someone feels extremely low levels of negative emotions, such as anxiety or fear, the individual is likely to have psychopathy and show antisocial behaviors [34]. Therefore, experiencing certain levels of negative emotions is beneficial and affect balance is considered a suitable emotional experience for learning.

Finally, when simultaneously considering PoE and affect balance, affect balance can significantly predict both behavioral and affective engagement, while PoE can only significantly predict behavioral engagement. Additionally, affect balance serves as a mediator in the relationship between PoE and L2 learning engagement. This further enriches the results of Liu and Wang [9], indicating that, in addition to L2 enjoyment and anxiety mediating the relationship between grit and language performance, affect balance also mediates the relationship between PoE and L2 learning engagement. The complete mediating effect of affect balance in the relationship between PoE and L2 affective engagement confirms the critical role of affect balance in affective engagement. It also demonstrates how affect balance can influence the impact of related personality variables on L2 affective engagement. The partial mediating effect of affect balance in the relationship between perseverance of effort and behavioral engagement suggests that affect balance can also play a role in behavioral engagement. Therefore, in the analysis of learning engagement, it is essential to consider personality variables such as perseverance of effort and incorporate emotion-related influencing factors. Specifically, a balanced state of negative and positive emotions is crucial for language learners’ behavioral and affective engagement. Thus, language educators need to have a comprehensive understanding of learners’ emotions, not only focusing on positive or negative emotions but also grasping learners’ emotional experience as a whole.

### 4.4. Limitations and Future Directions

Although this study has discovered the significant roles of general grit and affect balance in L2 learning engagement, as well as the differential correlational relationships between CoI and PoE with L2 learning engagement, and the mediating role of affect balance in the relationship between L2 learning engagement and PoE, there are several aspects worth considering in future research.

Firstly, most studies on the role of emotions in the L2 learning process adopt cross-sectional research designs and lack longitudinal research. Emotions in individual language learning experiences are dynamic and unstable. Although this study measured learners’ L2 learning emotions by requiring participants to recall their overall learning emotional experiences over the past month, there is still a certain time limitation. Therefore, future research could extend the time for tracking emotional changes and explore the characteristics of L2 emotions over more extended periods, examining their relationship with L2 academic achievements and engagement.

Secondly, categorizing emotions into positive and negative groups is a relatively binary approach to emotions. While valence is an essential variable in explaining differences in emotional activation [35], some argue that the unique effects of discrete emotions cannot be entirely explained by valence [36]. Therefore, future research could simultaneously consider multiple discrete emotions, such as shame, which has been less studied, and investigate how they, along with personality traits like grit, collectively influence L2 learning outcomes and engagement.

Lastly, a considerable amount of research focuses on L2 learning, with less emphasis on third language (L3) research [37]. While L3 learning shares similarities with L2 learning, it also has its unique characteristics. Hence, future research could further explore the roles of emotions and personality traits in the L3 learning process.

## Figures and Tables

**Table 1:** Descriptive statistics for variables.

	M ± SD
Behavioral engagement	5.35 ± 1.05
Affective engagement	5.05 ± 1.23
Consistency of interest	1.84 ± 0.79
Perseverance of effort	3.35 ± 0.74
Grit	2.59 ± 0.61
Negative emotions	2.40 ± 0.81
Positive emotions	3.47 ± 0.73
Affect balance	1.07 ± 1.35

**Table 2:** Correlation matrix of variables.

Variables	BE	AE	CoI	PoE	Grit	NE	PE	AB
BE	—							
AE	0.77 ***	—						
CoI	0.00	−0.01	—					
PoE	0.35 ***	0.28 ***	0.26 ***	—				
Grit	0.21 ***	0.18 ***	0.81 ***	0.78 ***	—			
NE	−0.26 ***	−0.41 ***	−0.22 ***	−0.18 ***	−0.25 ***	—		
PE	0.56 ***	0.70 ***	0.05	0.42 ***	0.29 ***	−0.54 ***	—	
AB	0.46 ***	0.63 ***	0.16 **	0.34 ***	0.31 **	−0.89 ***	0.86 ***	—

Note: BE: behavioral engagement, AE: affective engagement, CoI: consistency of interest, PoE: perseverance of effort, PE: positive emotion, NE: negative emotion, AB: affect balance. *** *p* < 0.001, ** *p* < 0.01.

## Data Availability

The data are available from the corresponding author upon reasonable request.
